# Supplementary material for: Effects of Methylprednisolone on Ventilator-Free Days in Mechanically Ventilated Patients with Acute Respiratory Distress Syndrome and COVID-19: A Retrospective Study
Source: J Clin Med. 2021 Feb 14;10(4):760. doi: 10.3390/jcm10040760 (PMC7917584; doi:10.3390/jcm10040760)
Supplement: Supplementary file 1 [file jcm-10-00760-s001.pdf]

Table S1. Univariate competing-risks regression analysis.

Variables	SHR (95% confidence interval)	p-value
**Methylprednisolone treatment, (refer: no)**	0.46 (0.25-0.85)	**0.013**
**Age, year**	1.03 (0.99-1.06)	**0.062**
**Male, n (%)**	1.18 (0.47-2.98)	0.73
**Body mass index, kg.m^{-2}**	1.02 (0.97-1.07)	0.51
**SOFA**	1.01 (0.96-1.25)	0.16
**SAPS II**	1.01 (0.99-1.03)	0.24
**Patients with comorbidities, (refer: no)**	1.19 (0.67-2.08)	0.55
**Comorbidities distribution, (refer: no)**		
Diabetes mellitus	0.79 (0.45-1.39)	0.41
Hypertension	1.44 (0.76-2.72)	0.26
Chronic kidney disease	2.16 (1.19-3.91)	**0.011**
Chronic artery disease	0.93 (0.37-2.34)	0.89
**Time from symptoms onset to ICU admission, day**	0.97 (0.90-1.04)	0.41
**Time from symptoms onset to intubation, day**	0.98 (0.91-1.04)	0.48
**Time from symptoms onset to Methylprednisolone administration, day**	1.00 (0.92-1.08)	0.99
**Vital signs on ICU admission**		
Temperature (max), °C	1.23 (0.95-1.73)	0.10
Heart rate, beats.min^{-1}	1.00 (0.99-1.01	0.27
Respiratory rate, breaths.min^{-1}	1.01 (0.98-1.04)	0.50
**Laboratory data on ICU admission**		
C-reactive protein, mg.L^{-1}	1.00 (0.99-1.001)	0.31
Leucocytes count, × 10^9.l^{-1}	0.98 (0.91-1.05)	0.59
Lymphocytes count, × 10^9.l^{-1}	1.73 (0.58-5.09)	0.32
Platelet count, × 10^9.l^{-1}	1.00 (0.99-1.002)	0.55
Procalcitonin, ng.mL^{-1}	1.01 (1.00-1.02)	**0.005**
International normalized ratio	0.84 (0.14-5.18)	0.85
Activated partial thromboplastin time; s	1.01 (0.98-1.04)	0.42
D-dimer, µg.mL^{-1}	0.86 (0.70-1.06)	0.17
Fibrinogen, g.L^{-1}	1.09 (0.94-1.26)	0.27
Ferritin, µg.L^{-1}	1.00 (0.99-1.00)	0.96
Interleukin 6, ng.L^{-1}	1.00 (0.99-1.00)	0.28
Alanine aminotransferase, IU.mL^{-1}	1.00 (0.99-1.01)	0.81
Aspartate aminotransferase, IU.mL^{-1}	1.00 (0.99-1.02)	0.23
Total bilirubin, µmol.L^{-1}	0.99 (0.97-1.01)	0.47
Creatinine, µmol.L^{-1}	1.00 (0.99-1.00)	**0.09**
PaO_2/FiO_2 ratio, mmHg	1.00 (0.99-1.00)	0.19
SaO_2, %	0.97 (0.93-1.00)	**0.08**
$PaCO_2$, mmHg	1.00 (0.98-1.03)	0.66
Lactate, mmol.L^{-1}	1.38 (0.98-1.93)	**0.06**
**Treatments (refer: no)**		
Vasopressor support	2.80 (1.46-5.39)	**0.002**
Prone position	3.08 (1.60-5.95)	**0.001**

Neuromuscular blocker agents	2.11 (1.12-3.98)	**0.021**
Renal replacement therapy	2.12 (0.72-6.27)	0.17
Tocilizumab	1.14 (0.69-1.89)	0.60
Hydroxychloroquine	1.13 (0.64-2.00)	0.67
Favipiravir	0.80 (0.42-1.51)	0.49
Lopinavir/ritonavir	1.11 (0.63-1.98)	0.71
**Ventilator parameters**		
Tidal volume, $mL.kg^{-1}$ IBW	1.07 (0.83-1.37)	0.61
Plateau pressure, cmH_2O	1.02 (0.95-1.10)	0.63
Positive end expiratory pressure, cmH_2O	1.00 (0.83-1.22)	0.93
Driving pressure, cmH_2O	1.01 (0.94-1.09)	0.72
Static respiratory compliance, $mL.cmH_2O^{-1}$	0.99 (0.95-1.03)	0.68

SOFA, Sequential Organ Failure Assessment; SAPS, Simplified Acute Physiology Score; PaO_2, arterial oxygen tension; $PaCO_2$, arterial CO_2 tension; FiO_2; Inspiratory oxygen fraction; SaO_2, arterial oxygen saturation; IBW, ideal body weight; ICU, intensive care unit; SHR, sub hazards ratio.

$p \leq 0.05$ was considered statistically significant.
